# 

**Published:** 1886-10

**Authors:** 


| | PAGE. |
|---|---|
| ORIGINAL COMMUNICATIONS—Avian Tuberculosis. An illustration of Amœbic Warfare. John Bland Sutton, F. R. C. S. | 329 |
| On Injuries of the Beak in Birds, and the Method of Repair. R. W. Shufeldt, M. D. | 357 |
| The Rôle of Microbes and their Chemical Products in the Causation of Disease. James Law, F. R. C. V. S. | 360 |
| "Barsati," or Atrophic Carcinoma. Richard W. Burke, V. S., A. V. D. | 368 |
| Phenomena and Evidence of Serpent Poison. G. Archie Stockwell, M. D. F. Z. S. | 377 |
| Thomas Walley, M. R. C. V. S. Principal Royal (Dick) Veterinary College, Edinburgh | 387 |
| U. S. Army Veterinarians. James A. Waugh, V. S. | 390 |
| EDITORIAL DEPARTMENT | 394 |
| CASE DEPARTMENT | 400 |
| PROCEEDINGS VETERINARY MEDICAL SOCIETIES | 405 |
| PROGRESS OF VETERINARY SCIENCE | 411 |
| REVIEWS | 414 |

